# Effects of different chlorhexidine pretreatments on adhesion of metal brackets *in vitro*

**DOI:** 10.1186/1746-160X-8-36

**Published:** 2012-12-28

**Authors:** Corinne Frey, Enver Yetkiner, Bogna Stawarczyk, Thomas Attin, Rengin Attin

**Affiliations:** 1 Private Practice, Winterthur, Switzerland; 2 Department of Orthodontics, University of Ege, Izmir, Turkey; 3 Department for Prosthodontics, University of Munich, Munich, Germany; 4 Clinic of Preventive Dentistry, Periodontology and Cariology, Center of Dental Medicine, University of Zurich, Zürich, Switzerland; 5 Department of Orthodontics and Pediatric Dentistry, University of Zurich, Zürich, Switzerland

**Keywords:** Chlorhexidine, Adhesion, Shear bond strength

## Abstract

**Objective:**

To investigate the effect of chlorhexidine applications in various forms and concentrations on adhesion and failure modes of metal brackets *in vitro*.

**Material and methods:**

Ninety bovine enamel specimens were allocated to six groups (n=15). Metal brackets were bonded on all specimens after chlorhexidine pre-treatments forming the following groups: (1) untreated specimens (control); (2) 40% varnish (EC40, Biodent BV, Netherlands), remnants removed with brushing mimicking patient cleaning; (3) 40% varnish (EC40), remnants removed with brushing mimicking professional cleaning; (4) 1% varnish (Cervitec Plus, Ivoclar vivadent, Schaan, Liechtenstein), remnants not removed; (5) brushed with% 1 gel (Corsodyl, GlaxoSmithKline, Münchenbuchsee, Germany), remnants not removed; (6) immersed in 0.07% mouthrinse (Corsodyl, GlaxoSmithKline, Münchenbuchsee, Germany), remnant not rinsed. Debonding of brackets was performed using a universal testing machine. Data were analysed using one-way ANOVA and post-hoc Scheffé test.

**Results:**

Group 4 performed significantly inferior than all the other groups and the control. Group 4 presented the highest number of adhesive failures at the enamel-resin interface whereas in other groups no failures at adhesive-resin interface was observed.

**Conclusion:**

Presence of chlorhexidine varnish prior to bracket bonding adversely affects adhesion. Concentration of chlorhexidine pre-treatment has no influence on shear bond strength.

## Introduction

Standard caries prevention measures based on mechanical plaque removal, non-cariogenic dietary habits and regular fluoride supplementation are often insufficient to prevent new lesions in orthodontic patients [1]. Following the formation of a highly colonized cariogenic micro-flora, neither tooth brushing nor increased fluoride delivery is capable of effectively preventing the demineralization process [1,2]. Thus, the use of an antimicrobial agent to suppress cariogenic bacteria, and thereby to inhibit the development of new caries lesions, seems to be a rational approach during orthodontic treatment [3,4].

Chlorhexidine (CHX) is the most potent documented antimicrobial against *Mutans streptococci* (MS), one of the most pronounced bacteria causing early enamel caries [1]. CHX is commonly delivered in forms of varnishes, gels and rinsing solutions, which also determine the mode of its effect. The persistence of bacterial suppression is related to contact time of CHX with intraoral tissues, its rate of release and concentration. Depending on these particular factors, CHX varnishes establish the most persistent reduction in MS followed by gels and mouthwashes [2]. Other than these delivery forms, innovative trials have been performed to add CHX into resins to have a constant rate of antibacterial release intra-orally [5-7]. However, the established rate of CHX release was not linear, rather decreasing rapidly following initial application [5,7]. Moreover, as the amount of CHX added was increased to keep the level of CHX release over minimum inhibitory levels, more unreacted monomers and additives were released from the adhesive resulting in an increase of voids within the resin, which weaken polymerisation features [6,8,9].

It can be anticipated that adhesion of brackets, a technique-sensitive application, might be prone to deteriorating influences of such pre-treatments that might interfere with the bonding procedure. Adsorption of CHX by the enamel and remnants of CHX that are expected to prolong antibacterial agent release over time might limit direct contact of the etching agent resulting in incomplete etching [10]. Numerous studies have investigated the possible effects of different CHX applications on the adhesion of brackets. CHX was tested in all three forms under following conditions: before acid etching without subsequent surface cleaning; after enamel etching mixed with bonding agent; after enamel etching alone, without additional bonding agent; after enamel etching prior to photo-polymerization of the bonding agent; after enamel etching following the photo-polymerization of the bonding agent and mixed with bonding agent on hydrophilic primer applied etched enamel [10-16]. No adverse effect was reported when CHX with low concentrations was applied prior to acid etching but controversial results were reported following applications after acid etching where only mixing CHX varnish with the bonding agent was agreed to be a safe application by the researchers [10,11]. On the other hand, there is no information in the literature investigating the possible effects of CHX application modes with various concentrations on adhesion of brackets. Moreover, there is no manufacturer instruction clarifying the appropriate mode of application and the state of bonding area prior to bracket placement, which is directly influential on persistence of bacterial suppression. Theoretically, adhesion might get affected by the presence of CHX on the enamel surface depending on its form and concentration [10,16]. To test this question, the aim of this present study was to investigate the influence of various pre-treatments with CHX applied according to manufacturer instructions on adhesion of brackets prior to bracket placement and observe the failure types following debonding. The hypothesis tested was that CHX pre-treatments that were removed from the surface prior to bonding procedures would have no adverse effect on the adhesion of metal brackets.

## Materials and methods

### Specimen preparation

Bovine incisors (N=90) stored in 0.5% chloramine solution at 4°C no longer than 6 months were initially cut from their roots. Enamel discs with a diameter of 6.6 mm were cut from the labial aspect of each tooth using a custom-made diamond-coated trephine bur (80 μm, Intensiv SA, Lugano-Grancia, Switzerland). The discs were then flattened from the bottom to approximately 2 mm in height (Struers, Birmensdorf). Pieces were randomly assigned to 6 groups and were embedded with their labial surfaces exposed in auto-polymerizing acrylic resin (Paladur, Heraeus Kulzer, Wehrheim, Germany) in cylindrical moulds. Embedded specimens were ground flat and polished with water-cooled carborundum discs (1200, 2400 and 4000 grit, Struers, Erkrat, Germany). The specimens were stored in distilled water (grade 3) until chlorhexidine pre-treatments and bonding procedures.

### Chlorhexidine Pre-Treatments

Group 1: Control, no pre-treatment applied.

Group 2: 40%CHX varnish was applied in one coat on air-dried surface, let set for 10 m, cleaned with 15 brushing strokes with 250 g load simulating tooth brushing of a patient.

Group 3: 40% CHX varnish was applied in one coat on air-dried surface, let set for 10 m, cleaned with a proxy brush until the surface was visibly free of any varnish remnants.

Group 4: 1% CHX varnish was applied in one coat on air-dried surface and let set for 60 s. No cleaning performed.

Group 5: 1% CHX Gel was used to brush teeth with 15 strokes, no cleaning performed.

Group 6: Specimen was immersed in 0.07% CHX mouth-rinse for 60 seconds and air-dried, no cleaning performed.

All pre-treatment applications of the respective products were conducted according to manufacturers’ instructions. The generic names, administration forms, chemical compositions and manufacturers of products are listed in Table 1.

**Table 1:** Generic names, administration forms, chemical compositions and manufacturers of products

**Product**	**Form**	**Chemical composition**	**Manufacturer**
EC 40	Varnish	Chlorhexidine Diacetate 35%	Biodent, Nijmegen,
		Sandarac Resin 27%	Netherlands
		Ethanol 38%	
Cervitec Plus	Varnish	Chlorhexidine Diacetate 1%	Ivoclar Vivadent,
		Thymol 1%	Schaan, Liechtenstein
		Ethanol, water, acrylate copolymer	
		Vinyl acetate copolymer	
Corsodyl	Gel	Chlorhexidine Digluconate 1%	GlaxoSmithKline,
		Isopropyl alcohol 4%	Bühl, Germany
		Non-Hazardous ingredients 95%	
Corsodyl	Mouthrinse	Chlorhexidine Digluconate 0.07%	GlaxoSmithKline,
		Sodium Fluoride 0.06%	Bühl, Germany
		Ethanol 5.5%	
		Non-significant components 94.37%	

bis-GMA: Bis-phenol-A-glycidylmethacrylate; TEGDMA: Triethyleneglycol dimethacrylate.

### Application of brackets

Following each pre-treatment, the specimens were etched with 37% H_3_PO_4_ (Orbis Dental, Münster, Germany) for 30 s, rinsed with water for 30 s and air-dried for 10 s. Metal brackets with 8.71 mm^2^ laser-structured bases for central lower incisors (Discovery, slot 0.56·0.76 mm / 22·30, Dentaurum, Ispringen, Germany) were bonded on specimens using a photo-polymerized conventional adhesive (Transbond XT, 3M Unitek, Monrovia, USA). Brackets were placed on enamel surfaces under standard load of 500 g. Excess resins were removed with foam pellets under 4.8× magnification. Photo-polymerization was achieved using LED polymerization device for 15 seconds from incisal, gingival, mesial and distal directions (Epilar Freelight II LED, 3MESPE, Seefeld, Germany; Output=1000 mW/cm2).

### Shear bond strength testing

Specimens were stored in distilled water (grade 3) for 24 hours at 37°C after bracket bonding. Shear bond strength (SBS) was tested using a Universal Testing Machine (Z010, Zwick, Ulm, Germany). A stainless steel rod with a chisel configuration was used for debonding (cross-head speed: 1 mm/min). Load at failure was recorded and bond strength values were calculated according to the following equation: S=F/A, where S is bond strength (MPa), F is load at failure (N), and A representing adhesive area (mm^2^).

### Failure analysis

The debonded area was examined under a stereomicroscope at 40x magnification (M3B, Wild, Heerbrugg, Switzerland). Failure was considered: adhesive - if the cement/resin was dislodged from enamel; cohesive in the cement/resin - if the fracture occurred only in the cement/resin; cohesive in enamel – if the fracture occurred only in the enamel.

### Data analysis

Data were analysed using SPSS software for Windows Version 20 (SPSS, Chicago, IL, USA). Mean values, standard deviations, minimum, maximum and 95% confidence interval (95% CI) were calculated. One-way ANOVA followed by a post-hoc Scheffé-test were applied to find differences in shear bond strength between the experimental treatments. Results with p-value smaller than 5% were interpreted as significant.

Additionally, failure types were classified and the relative frequencies of failure mode in each tested group were computed together with the corresponding 95% confidence interval.

## Results

**Figure 1:**
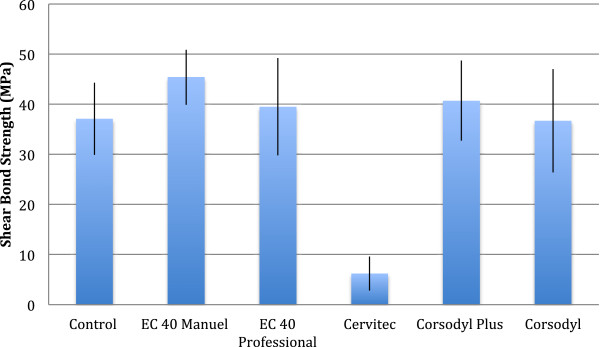
Mean shear bond strengths and standard deviations.

1% CHX varnish which was not removed prior to bracket bonding (6.2 ± 3.4 MPa) presented significantly lower shear bond strengths compared to the other groups (p<0.001) and the control. All other forms of applications presented similar bond strengths and showed no difference with the control. The results of the descriptive statistics (mean, 95% confidence interval, standard deviation, minimum and maximum) for shear bond strength for each group are presented in Table 2 and Figure 1.

**Table 2:** Mean shear bond strength, standard deviations (SD), 95% confidence interval (CI) for mean, minimum (Min), maximum (Max) values for each group

**Groups**	**Mean ± SD (MPa)**	**95% CI**	**Min**	**Max**
Control	37.1 ± 7.2 A	33.0 - 41.2	23.2	49.1
EC 40 Manuel	45.4 ± 5.5 A	42.3 - 48.6	34.9	56.5
EC 40 Professional	39.5 ± 9.7 A	34.0 - 44.9	19.9	51.4
Cervitec	6.2 ± 3.4 B	4.3 - 8.2	0.8	13.3
Corsodyl Plus	40.7 ± 8.0 A	36.2 - 45.1	28.5	56.1
Corsodyl	36.7 ± 10.3 A	30.9 - 42.5	16.6	54.2

Means that are not significantly different are marked with the same capital letters.

No adhesive failure at the resin-enamel interface was observed except 1% CHX varnish group. The other groups presented mainly adhesive failures at the resin-bracket interface (80-93%). Relative frequencies (%) with 95%-confidence interval in brackets of different failure types are presented in Table 3.

**Table 3:** Relative frequencies (%) with 95%-confidence interval in brackets of different failure types

	**Groups**					
**Failure location**	**Control**	**EC 40 Manuel**	**EC 40 professional**	**Cervitec**	**Corsodyl plus**	**Corsody**
Resin-bracket	93 (68-100)	87 (59-99)	87 (59-99)	Ø	87 (59-99)	93 (68-100)
Mixed	7 (0-32)	13 (1-41)	13 (1-41)	20 (4-49)	13 (1-41)	7 (0-32)
Resin-enamel	Ø	Ø	Ø	80 (51-96)	Ø	Ø

## Discussion

This study aimed to investigate the influence of various pre-treatments with CHX on adhesion of brackets prior to acid etching and the failure types following debonding. The results showed that all CHX forms except 1% CHX varnish which was not removed prior to bracket bonding could be used safely prior to bracket placement when used according to manufacturers’ instructions.

In the present study, three different forms of CHX formulations were tested with concentrations ranging from 1% to 40%. Although 1% CHX varnish applied specimens presented very low bond strengths, brackets in 1% CHX gel and 0.07% CHX mouthrinse groups provided adhesion well above the clinically accepted value. Similarly application of 40% CHX varnish had no adverse effect on the adhesion of brackets. These findings indicated that the concentration of CHX applied prior to acid etching had no effect on the bonding properties of metal brackets on enamel.

In the present experimental set-up the CHX application procedures were performed according to the recommended manufacturers’ instructions. EC 40, the 40% CHX varnish was applied and let set for 10 m and the remaining product on the surface was removed either with tooth-brushing of 15 strokes mimicking patients’ cleaning or with a proxy brush until all visible remnants were removed mimicking professional cleaning. On the other hand, Cervitec, the 1% varnish was not removed after its application and a layer of CHX remained prior to the bonding procedures. Similarly the Corsodyl gel and mouthrinse, the 1% gel and 0.07% mouthrinse, respectively were not rinsed or cleaned following their application protocols. Even though the cleaning of the bonding surface would be expected, these modes of applications were advocated by the manufacturers with the idea of prolonging the CHX release from the affected surface, in other words the bacteria inhibiting effect. However, the remaining layer of Cervitec seemed to play an isolating role on the enamel prohibiting the effective bonding procedure resulting in very low shear bond strengths. This was further corroborated when the location of failure types were examined where only Cervitec group presented adhesive failures at the resin-enamel interface. There are two possible explanations of these results; the relatively thick layer remaining on the enamel might have prevented the acid to reach the calcified tissues underneath or this layer might have acted as a physical barrier for the resin to penetrate enamel. On the other hand, in the two other groups where CHX was not rinsed or removed, this was not the case. Specimens brushed with the 1% CHX gel, Corsodyl gel or the specimens that were immersed in the 0.07 CHX mouthrinse, Corsodyl mouthwash provided bond strengths well above the clinically accepted value and presented no adhesive failures at the resin- enamel interface. This indicated that there was no interference during enamel etching or resin impregnation resulting in greater adhesive forces between the resin and enamel. Nevertheless, it should be pointed out that the remaining layer of CHX on the enamel surface in these two groups were much thinner, probably due to lower viscosity of the products and the mode of application. With these aspects in mind, it can be suggested that varnish forms of CHX applied should be removed mechanically following application in case of planned bonding procedures.

Most of the studies testing the effects of CHX applications on bracket adhesion have used the antibacterial agent following etching of enamel with a similar principle to cavity disinfection [10-14,16]. Parallel to this idea, CHX was added to the bonding agent and applied on the etched enamel surface and photo-polymerized in order to prolong its release. These modes of applications showed no adverse effect on the bonding procedure when the bonding agent had primary contact with the etched enamel and photo-polymerized when mixed with the CHX content [10-13]. On the other hand, adhesion was not achieved when CHX varnish was applied on the cured primer surface and not cured [10,13]. The efficacy of these applications in terms of prolonged CHX release, amount of total CHX released and the effects on physical properties of the resin cement were not questioned in vitro or in vivo. These aspects were investigated in dentin bonding studies where a linear CHX release was not observed [5-7]. In the present study, CHX was administered prior to enamel etching aiming to simulate its use as suppressing MS and changing the intraoral flora in the favour of non-cariogenic bacteria. This mode of administration has been widely accepted in studies aiming prevention of decalcification during orthodontic treatment even though recolonization of bacteria remains an unresolved issue.

## Conclusion

Different concentrations of chlorhexidine products can be applied safely prior to orthodontic bracket placement. However, remnants of chlorhexidine, when applied in the varnish form, should be removed in order to prevent compromised bond strength of brackets. In the case of the 40% varnish, removal of varnish is done professionally. For this kind of varnish, even manual brushing after application seems to be sufficient to remove remnants effectively.

## Abbreviations

CHX: Chlorhexidine; MS: Mutans Streptococci.

## Competing interests

The authors declare that they have no competing interests.

## Authors’ contributions

BS carried out the laboratory testing procedures, CF and EY drafted the manuscript, TA participated in the design of the study and helped to draft the manuscript, RA conceived of the study, participated in its design and helped to draft the manuscript. All authors read and approved the final manuscript.
